# Cardiolipin Synthesis and Outer Membrane Localization Are Required for *Shigella flexneri* Virulence

**DOI:** 10.1128/mBio.01199-17

**Published:** 2017-08-29

**Authors:** Rachael M. Rossi, Lauren Yum, Hervé Agaisse, Shelley M. Payne

**Affiliations:** a Department of Molecular Biosciences and Institute for Cellular and Molecular Biology, The University of Texas at Austin, Austin, Texas, USA; b Department of Microbiology, Immunology, and Cancer Biology, University of Virginia School of Medicine, Charlottesville, Virginia, USA; Princeton University

**Keywords:** IcsA, *Shigella flexneri*, cardiolipin, cell-cell spread, outer membrane

## Abstract

Cardiolipin, an anionic phospholipid that resides at the poles of the inner and outer membranes, is synthesized primarily by the putative cardiolipin synthase ClsA in *Shigella flexneri*. An *S. flexneri clsA* mutant had no cardiolipin detected within its membrane, grew normally *in vitro*, and invaded cultured epithelial cells, but it failed to form plaques in epithelial cell monolayers, indicating that cardiolipin is required for virulence. The *clsA* mutant was initially motile within the host cell cytoplasm but formed filaments and lost motility during replication and failed to spread efficiently to neighboring cells. Mutation of *pbgA*, which encodes the transporter for cardiolipin from the inner membrane to the outer membrane, also resulted in loss of plaque formation. The *S. flexneri pbgA* mutant had normal levels of cardiolipin in the inner membrane, but no cardiolipin was detected in the outer membrane. The *pbgA* mutant invaded and replicated normally within cultured epithelial cells but failed to localize the actin polymerization protein IcsA properly on the bacterial surface and was unable to spread to neighboring cells. The *clsA* mutant, but not the *pbgA* mutant, had increased phosphatidylglycerol in the outer membrane. This appeared to compensate partially for the loss of cardiolipin in the outer membrane, allowing some IcsA localization in the outer membrane of the *clsA* mutant. We propose a dual function for cardiolipin in *S. flexneri* pathogenesis. In the inner membrane, cardiolipin is essential for proper cell division during intracellular growth. In the outer membrane, cardiolipin facilitates proper presentation of IcsA on the bacterial surface.

## IMPORTANCE

The human pathogen *Shigella flexneri* causes bacterial dysentery by invading colonic epithelial cells, rapidly multiplying within their cytoplasm, and then spreading intercellularly to neighboring cells. Worldwide, *Shigella* spp. infect hundreds of millions of people annually, with fatality rates up to 15%. Antibiotic treatment of *Shigella* infections is compromised by increasing antibiotic resistance, and there is no approved vaccine to prevent future infections. This has created a growing need to understand *Shigella* pathogenesis and identify new targets for antimicrobial therapeutics. Here we show a previously unknown role of phospholipids in *S. flexneri* pathogenesis. We demonstrate that cardiolipin is required in the outer membrane for proper surface localization of IcsA and in the inner membrane for cell division during growth in the host cell cytoplasm.

## INTRODUCTION

*Shigella flexneri* is an intracellular pathogen that causes bacterial dysentery in humans (1). To cause disease, *S. flexneri* must invade colonic epithelial cells (2), proliferate and move within the cytoplasm (3), and then spread intercellularly by penetrating neighboring cells (4). The disease symptoms, which include bloody diarrhea and painful cramping, are the results of damage to the colonic epithelial layer by *S. flexneri* and by the inflammatory immune response of the host. *Shigella* spp. are closely related to *Escherichia coli* but encode a number of virulence determinants, many of which are encoded on a large plasmid (5). Virulence proteins include components of a Type III secretion system (T3SS) that injects effector proteins into the cytoplasm of the epithelial cells to initiate uptake of the bacteria and alter host cell responses (6). The subsequent spread to adjacent epithelial cells requires intracellular replication, movement mediated by cytoplasmic host actin (7), and reactivation of the T3SS (8–10).

*S. flexneri*, which is nonmotile, uses actin-based propulsion to move through the host cell cytoplasm and protrude into the neighboring cell. Actin-based motility requires proper localization of the virulence protein IcsA on the bacterial surface (11, 12). IcsA mutants are invasive and replicate within epithelial cells but cannot spread to adjacent cells (13). IcsA is secreted through the inner membrane via the Sec system (14) and requires the periplasmic chaperone proteins DegP, Skp, and SurA for localization to the outer membrane (15, 16). IcsA is a member of the autotransporter protein family (17); its carboxy-terminus domain forms a beta barrel channel to mediate the transfer of its amino-terminal passenger proteinase domain across the outer membrane (18). Once IcsA is properly oriented on the bacterial surface, the amino-terminal domain recruits phosphorylated neural Wiskott-Aldrich syndrome protein (N-WASP) (19, 20), which in turn recruits the Arp 2/3 complex that activates actin polymerization to propel the bacteria (21). The localization of IcsA on the surface of *S. flexneri* is to the old pole of the bacterial cell (11); however, the exact mechanism for unipolar targeting of IcsA remains unknown (22). Previous studies have shown that mutations that alter the O-antigen chain length of *S. flexneri* lipopolysaccharide (LPS) are associated with disruption of IcsA localization (23). An increase in O-antigen length can shield IcsA to inhibit its function (24), whereas decreased O-antigen chain length results in a uniform distribution of IcsA on the surface (25).

*S. flexneri*, like other Gram-negative bacteria, has an asymmetric outer membrane in which the lipid A portion of the LPS forms the outer leaflet, while the inner leaflet is made up of phospholipids (26). Both leaflets of the inner membrane are phospholipid. In *S. flexneri*, mutation of *vpsC*, a component of the Mla pathway (27), causes an accumulation of phospholipids in the outer leaflet of the outer membrane and remodeling of the lipid A species from hexa-acylated to hepta-acylated and results in impaired intercellular spread (28). Active remodeling of lipid A species during infection has been reported for Gram-negative pathogens, and these alterations allow the pathogens to evade the host immune response and protect themselves from environmental stress (29). However, it is not known whether the effects of *vpsC* mutation on *S. flexneri* virulence are due to changes in lipid A or the phospholipids in the outer membrane. In general, relatively little is known of the effects of specific phospholipids on bacterial pathogenesis.

During growth in exponential phase, phosphoethanolamine (PE) is the major phospholipid in the membrane of *E. coli*, representing almost 80% of the total phospholipids (30). The anionic phospholipids phosphatidylglycerol (PG) and cardiolipin account for about 18% and 2.5%, respectively (30). In the family *Enterobacteriaceae*, cardiolipin is synthesized within the inner membrane by ClsA, ClsB, and ClsC (30–32), and a portion of the cardiolipin is transported to the outer membrane by PbgA (33–35). Cardiolipin is a large anionic glycerol phospholipid composed of four large acyl chains connected by a small glycerol head group (36), giving it a conical shape. This shape allows it to accumulate at membrane regions that have negative curvature (37), including the negative membrane curvature regions of the inner mitochondrial matrix where cardiolipin was first identified (38). In *E. coli*, cardiolipin appears to localize to the poles of the bacterial inner leaflets of both the inner and outer membranes (39). Cardiolipin has been shown to play roles in both the localization and activity of electron transport proteins both in the mitochondria (36) and in the inner membrane of *E. coli* (40). Cardiolipin has also been shown to be important for localization or activity of proteins required for cell division (41) and osmotic stress response (42) in *E. coli*. In the absence of cardiolipin, the anionic phospholipid PG, which shares the same glycerol head group as cardiolipin, will localize to the bacterial poles (37) and interact (43) with proteins in a manner similar to that of cardiolipin, which has complicated studying the role of cardiolipin in bacterial membranes.

In this study, we determined that *clsA* encodes the major cardiolipin synthase and *pgbA* encodes the phospholipid transporter in *S. flexneri*. Both of these genes were required for *S. flexneri* plaque formation but acted at different points in the virulence pathway. This study demonstrates a role for phospholipids, specifically cardiolipin, in *S. flexneri* pathogenesis, and provides a model for their contribution to IcsA localization to the bacterial surface.

## RESULTS

### *S. flexneri* cardiolipin is synthesized primarily by ClsA.

**FIG 1:**
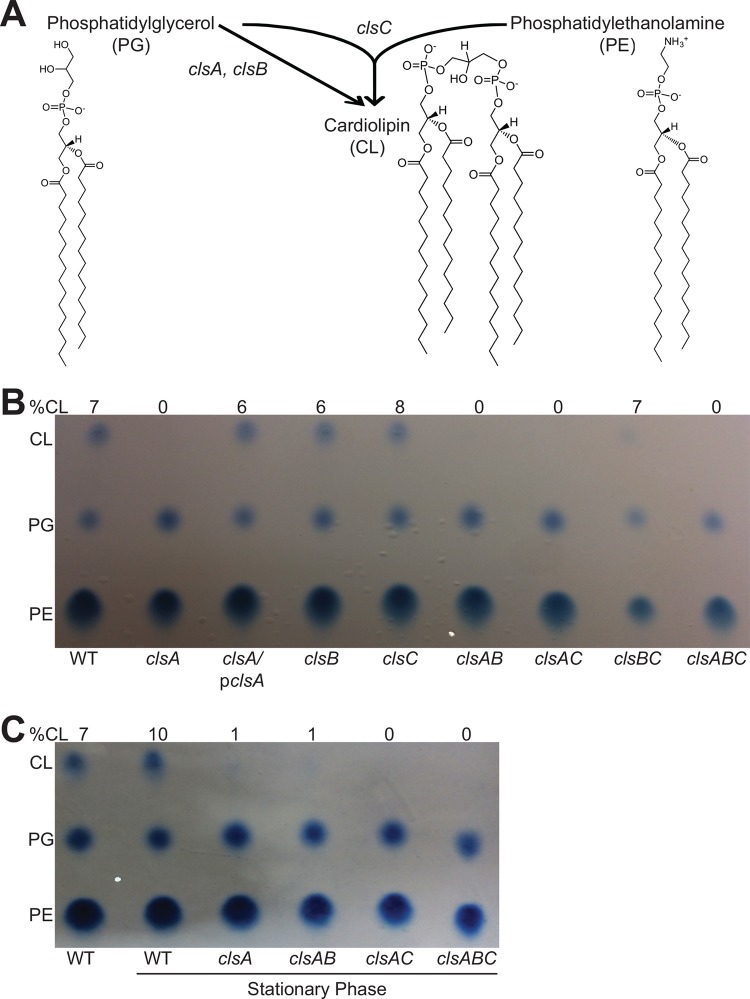
*clsA* is the major cardiolipin synthase of *S. flexneri*. (A) Schematic of *E. coli* cardiolipin synthesis (30–32). The chemical structures of phosphatidylglycerol (PG), phosphatidylethanolamine (PE), and cardiolipin (CL) were produced using ChemDraw (PerkinElmer). (B) TLC analysis of total *S. flexneri* membrane phospholipids of the wild type (WT) and cardiolipin synthesis mutants. Bacteria were grown to mid-log phase, and phospholipids were extracted and separated by TLC. The percentage of cardiolipin in the sample is indicated above each lane. p*clsA*, plasmid expressing *clsA*. (C) Bacteria were grown into stationary phase (OD_650_ of ~2.0); phospholipids were then extracted and separated by TLC. The percentage of cardiolipin in the sample is indicated above each lane. Phospholipids were visualized using molybdenum blue spray reagent (Sigma).

In *E. coli*, cardiolipin is synthesized by *clsA*, *clsB*, and *clsC*, which either condense two phosphatidylglycerol molecules (*clsA* and *clsB*) (30, 31) or condense phosphatidylglycerol and phosphatidylethanolamine molecules (*clsC*) (32) to produce cardiolipin (Fig. 1A). *S. flexneri*’s genome contains genes *cls*, *ybhO*, and *ymdC* with homology to *E. coli clsA*, *clsB*, and *clsC*, respectively, which we have renamed to match the *E. coli* gene nomenclature. To determine the contribution of each predicted *S. flexneri* cardiolipin synthase, we constructed individual deletion mutants and examined their phospholipid composition using Bligh-Dyer phospholipid isolation and subsequent thin-layer chromatography (TLC) separation for visualization (44). Compared to the wild type (WT), which has approximately 7% cardiolipin, deletion of *clsA* resulted in the loss of detected cardiolipin and an increase in the level of PG in the membrane of *S. flexneri* during exponential growth (Fig. 1B). The synthesis of cardiolipin was restored to near-wild-type levels by introducing *clsA* on a plasmid. Complementation with *clsA* also reduced PG levels to the wild-type levels. In contrast, the deletion of *clsB* and *clsC* had no effect on cardiolipin levels under these conditions, which were 6% and 8% of total phospholipids, respectively. This suggests that ClsA is the major cardiolipin synthase enzyme of *S. flexneri*.

### *S. flexneri* ClsC contributes to stationary phase cardiolipin synthesis.

Previous studies have shown that *E. coli* produces higher levels of cardiolipin during growth in stationary phase (45). Therefore, we extracted and separated phospholipids from *S. flexneri* grown to stationary phase. We found that the proportion of cardiolipin in *S. flexneri*’s membrane increased from 7% to 10% in stationary phase (Fig. 1C). Interestingly, the *clsA* mutant showed detected levels of cardiolipin (approximately 1%) during stationary phase, indicating that an additional cardiolipin synthase(s) is active. To determine which cardiolipin synthase is active during stationary growth, we assessed the phospholipid levels in the cardiolipin synthase double mutants and found that the *clsA clsC* double mutant lacked cardiolipin within its membrane during stationary-phase growth (Fig. 1C), indicating that ClsC is an active cardiolipin synthase during stationary phase.

### Cardiolipin localizes to both the inner and outer membranes of *S. flexneri*.

**FIG 2:**
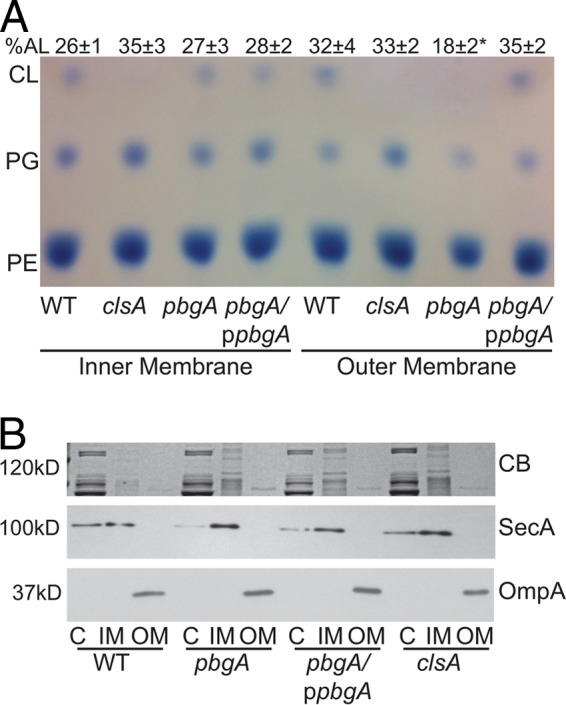
*S. flexneri* requires *pbgA* for localization of cardiolipin to its outer membrane. TLC analysis of *S. flexneri* inner and outer membrane phospholipids. (A) Inner and outer membrane composition analysis of *pbgA* and *clsA* mutants. Bacteria were grown to mid-log phase, and bacterial membranes were separated by solubilization in Sarkosyl. The phospholipids were then extracted, spotted for separation by TLC, and visualized using molybdenum blue spray reagent (Sigma). The phospholipids include phosphatidylglycerol (PG), phosphatidylethanolamine (PE), and cardiolipin (CL). The percentage of anionic phospholipids (AL) in the sample is indicated above each lane, and means ± standard deviations of three experiments are shown. The value that is significantly different (*P* < 0.05) from the value for the outer membrane of the wild type by Student’s *t* test is indicated by an asterisk. (B) Confirmation of clean inner and outer membrane fractions. Bacteria were grown to mid-log phase, and the bacterial membranes were separated by solubilization using Sarkosyl. Proteins were resolved by (10%) SDS-PAGE and stained with Coomassie blue (CB) or immunoblotted using either polyclonal anti-SecA or polyclonal anti-OmpA. The molecular masses (in kilodaltons) of molecular mass markers are shown to the left of the gels. p*pbgA*, plasmid expressing *pbgA*; C, cytoplasm; IM, inner membrane; OM, outer membrane.

To determine whether cardiolipin is found in both the inner and outer membranes of *S. flexneri*, we fractionated the inner and outer membranes using Sarkosyl solubilization and assessed phospholipid composition by thin-layer chromatography. Cardiolipin was found in both the inner and outer membranes of *S. flexneri*, and the phospholipid distributions in the inner and outer membranes were similar (Fig. 2A). To confirm clean separation of the inner and outer membranes, samples of cell fractions were analyzed by SDS-PAGE and Western blotting; the inner membrane protein SecA was not detected in the outer membrane fractions, and the outer membrane protein OmpA was found only in the outer membrane fractions (Fig. 2B). Analysis of the *clsA* mutant showed that the inner and outer membranes had similar phospholipid profiles, and both membranes had increased levels of PG in the absence of detected cardiolipin compared to the wild type. Wild-type levels of cardiolipin and PG in both the inner and outer membranes were restored by complementation with *clsA* on a plasmid.

### Cardiolipin transport to the outer membrane requires PbgA.

In *Salmonella enterica* serotype Typhimurium, activation of PhoPQ induces the expression of the *pbgA* gene, which encodes the PbgA protein that is responsible for transporting cardiolipin to the outer membrane, a requirement for maintaining outer membrane integrity during growth in the host cells (34, 46). Complete deletion of *pbgA* in *E. coli* and *S*. Typhimurium is lethal; however, mutants with a deletion of the C-terminal periplasmic portion of PbgA were viable but lacked cardiolipin transport to the outer membrane (33). Therefore, we made a similar deletion of the C terminus of the *S. flexneri pbgA* homolog (*yejM*) to determine the effects of eliminating cardiolipin from the outer membrane. Membrane fractionation and analysis of the phospholipids confirmed that the *S. flexneri pbgA* mutant lacked cardiolipin in the outer membrane (Fig. 2A), while the phospholipid composition of the inner membrane of the *pbgA* mutant looked identical to that of the wild type (Fig. 2A). The *clsA* mutant did not have cardiolipin in the outer membrane but had increased PG, maintaining the proportion of anionic lipids that make up the outer membrane similar to that of the wild type, approximately 32%. In contrast, because the *pbgA* mutant did not have increased PG levels in the outer membrane, the outer membrane anionic lipid percentage significantly decreased from wild type to only 18%. Together, these results suggest that *S. flexneri* PbgA, like the *S*. Typhimurium homolog, is responsible for transporting cardiolipin and some PG to the outer membrane and maintaining the normal levels of anionic phospholipids in the outer membrane.

### Cardiolipin synthesis is required for plaque formation.

**FIG 3:**
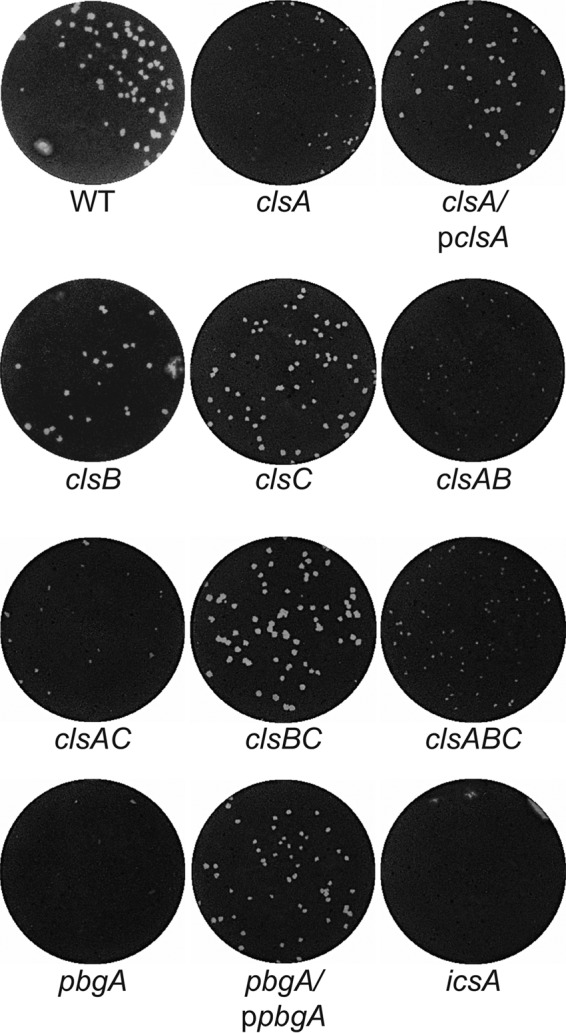
*clsA* and *pbgA* are required for *S. flexneri* plaque formation. Confluent monolayers of Henle cells were infected with approximately 10^4^ CFU of bacteria. Monolayers were stained and photographed after 72 h to visualize plaque formation.

To determine the role of cardiolipin in *S. flexneri* pathogenesis, we performed plaque assays (47) with each of the cardiolipin synthesis mutants. Plaque formation required invasion of the monolayer, intracellular replication, and spread to the adjacent cells. After 72 h, the *clsA* mutant formed pinpoint plaques compared to the WT, and plaque formation was complemented by *clsA* on a plasmid (Fig. 3). Because ClsC contributed to cardiolipin synthesis in stationary phase, it was possible that the ClsB or ClsC was expressed in the intracellular environment and contributed sufficient cardiolipin to support formation of very small plaques. Both *clsB* and *clsC* were induced approximately 10-fold in intracellular bacteria (see Fig. S1 in the supplemental material). However, mutations in *clsB* and *clsC* did not affect *S. flexneri* plaque formation (Fig. 3), indicating that cardiolipin synthesis by ClsA is required for wild-type plaque formation and that ClsB and ClsC do not synthesize sufficient cardiolipin in intracellular bacteria to compensate fully for the loss of *clsA*.

10.1128/mBio.01199-17.2 FIG S1  mRNA levels of *clsB* and *clsC* increase during intracellular growth as determined by quantitative real-time PCR. Bacteria were subcultured 1:100 into LB plus 0.01% DOC and grown to mid-log phase. Cultures were divided, RNA from approximately 10^8^ CFU was isolated and used to determine extracellular message levels, and semiconfluent Henle-407 monolayers were infected with approximately 10^8^ CFU. RNA was isolated 4 h postinfection and used to determine intracellular mRNA levels. Threshold cycle (*C*_*T*_) values were normalized against those for *accD*, analysis was performed using the ΔΔ*C*_*T*_ approach and are shown relative to the extracellular level set at 1. Values that are significantly different (*P* < 0.05) from the extracellular expression levels by Student’s *t* test are indicated by an asterisk. Download FIG S1, DOCX file, 0.2 MB. Copyright © 2017 Rossi et al. 2017 Rossi et al. This content is distributed under the terms of the Creative Commons Attribution 4.0 International license.

### PbgA, the transporter of cardiolipin to the outer membrane, is required for *S. flexneri* plaque formation.

To determine whether cardiolipin is required in the outer membrane of *S. flexneri* for virulence, the *pbgA* mutant was tested for plaque formation. The *pbgA* mutant was unable to form plaques, and plaque formation was restored by full-length *pbgA* on a plasmid (Fig. 3). This indicates that transport of cardiolipin to the outer membrane is critical for plaque formation. The *clsA* and *pbgA* mutants were further characterized to determine more precisely the roles of cardiolipin in *S. flexneri* virulence.

### Cardiolipin synthesis and transport do not contribute to *S. flexneri* membrane integrity.

Previous studies have shown that a disruption in outer membrane integrity inhibits *S. flexneri* plaque formation (28). To determine whether the defect in plaque formation by the *clsA* and *pbgA* mutants is the result of reduced outer membrane integrity due to lack of cardiolipin, we assessed the mutant for increased sensitivity to sodium deoxycholate (DOC), an ionic bile acid that disrupts the membrane. The *clsA* and *pbgA* mutants grew similarly to the wild type, with an average doubling time of 41 min, both in the presence (Fig. S2B) and absence (Fig. S2A) of DOC. In contrast, the *vpsC* mutant, which has compromised outer membrane stability (28), was inhibited in the presence of DOC. This suggests that cardiolipin within the outer membrane of *S. flexneri* is not playing a structural role in the integrity of the outer membrane of *S. flexneri*.

10.1128/mBio.01199-17.3 FIG S2  *clsA* and *pbgA* mutant strains do not exhibit growth sensitivity to DOC. (A and B) Bacteria were subcultured 1:100 into LB (A) or LB containing 0.1% DOC (B) and grown into stationary phase. Data shown are representative of three biological replicates. Download FIG S2, DOCX file, 0.7 MB. Copyright © 2017 Rossi et al. 2017 Rossi et al. This content is distributed under the terms of the Creative Commons Attribution 4.0 International license.

### Cardiolipin synthesis and transport are not required for *S. flexneri* cellular invasion and intracellular replication.

The initial stage of *S. flexneri* virulence requires the bacteria to invade colonic epithelial cells (2) followed by intracellular replication (48). A requirement for cardiolipin for either of these processes would result in reduced plaque formation. Therefore, we compared the *clsA* and *pbgA* mutants to the wild type and found that neither mutant had a significant defect in invasion compared to the wild-type parental strain (Table S1). Infection rates were determined for cells grown in the presence and absence of DOC, since previous work (49) had shown that DOC increased virulence protein secretion and infectivity of wild-type *Shigella*. To assess intracellular replication, we isolated intracellular bacteria at 60 and 180 min postinfection and determined their doubling time. We found that compared to the WT, neither the *clsA* nor *pbgA* mutant had a significant defect in intracellular replication (Table S1). Together, these data indicate that cardiolipin is not required for Henle cell invasion and intracellular replication during the first 3 h of infection.

10.1128/mBio.01199-17.4 TABLE S1  Invasion and intracellular growth rates of *clsA* and *pbgA* mutants. Superscript *a* indicates the percentage of Henle cells that contained three or more intracellular bacteria. The data represent mean values of three biological replicates with standard deviations. Superscript *b* indicates the intracellular doubling time of bacteria between 1 and 3 h of intracellular growth. The numbers of intracellular bacteria were determined by lysing Henle monolayers, followed by plating of the lysate dilutions. Data represent the mean values of three biological replicates and standard deviations. Compared to the wild type, none of the mutants had a statistical difference in invasion or intracellular doubling time by Student’s *t* test (*P* < 0.05). Download TABLE S1, DOCX file, 0.1 MB. Copyright © 2017 Rossi et al. 2017 Rossi et al. This content is distributed under the terms of the Creative Commons Attribution 4.0 International license.

### *S. flexneri* intercellular spread requires cardiolipin synthesis and transport to the outer membrane.

**FIG 4:**
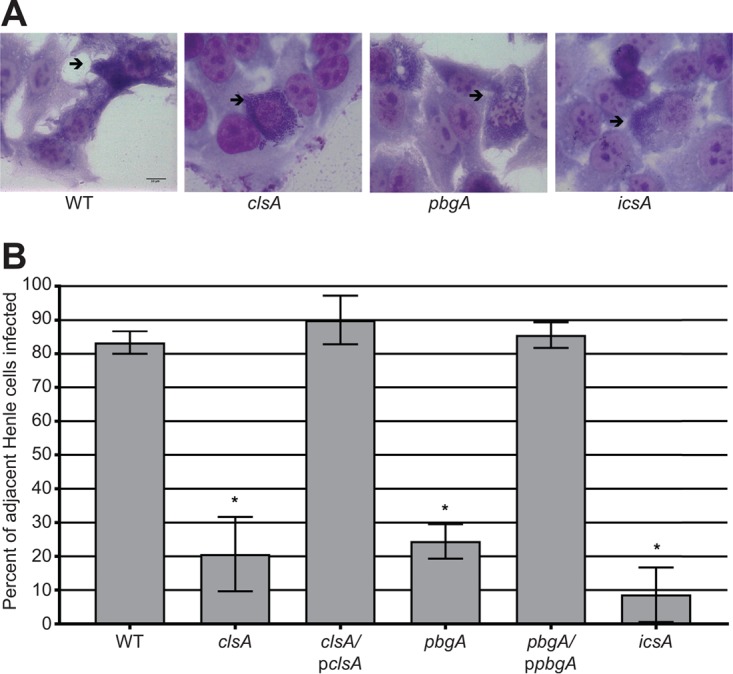
Cardiolipin is required for *S. flexneri* intercellular spread. Semiconfluent Henle monolayers were infected with approximately 10^7^ CFU of bacteria. Monolayers were stained after 4 h, and intercellular spread was visualized by bright-field microscopy. (A) Micrographs of intercellular spread by WT *S. flexneri* and *clsA*, *pbgA*, and *icsA* mutants. Black arrows point to primary infected Henle cells. (B) Graphical representation of *S. flexneri* intercellular spread. One hundred infected Henle cells were counted positive for spread if the surrounding Henle cells were also infected. Values are means ± standard deviations (error bars) for three biological replicates. Values that are significantly different (*P*  < 0.05) from the value for the wild type by Student’s *t* test are indicated by an asterisk.

The lack of an effect of cardiolipin on invasion and intracellular replication suggested that the plaque defect was due to lack of cell-to-cell spread of the bacteria (50). To determine the role of cardiolipin in *S. flexneri* intercellular spread, we determined the percentage of primary Henle cell infections resulting in spread to neighboring cells after 4 h, using a cell-to-cell spread assay (28). Primary infected Henle cells were identified as cells containing large numbers of bacteria, indicating intracellular replication (Fig. 4A). Infective centers were scored positive for spread if one or more neighboring cells had three or more intracellular bacteria. After 4 h, wild-type *S. flexneri* had spread from >80% of the initially infected cells to neighboring cells (Fig. 4B). The *clsA* and *pbgA* mutants, however, had significant defects in intercellular spread with spread rates of only 20 and 24%, respectively. These rates mimicked the rate of an *icsA* mutant, which cannot spread (13). These results suggest that the defect in plaque formation of both the *clsA* and *pbgA* mutants is their inability to spread intercellularly.

### Synthesis and transport of cardiolipin to the outer membrane of *S. flexneri* are required for intercellular dissemination in HT-29 cells.

**FIG 5:**
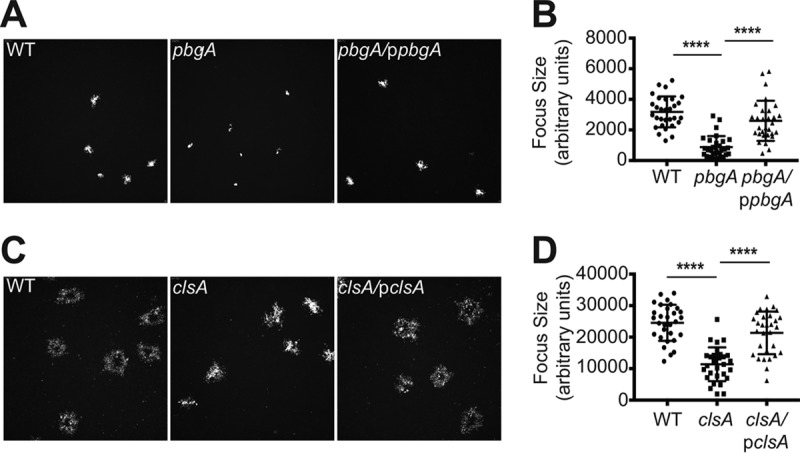
Quantification of *S. flexneri* dissemination in HT-29 cells. (A) Low-magnification image of WT and *pbgA* mutant focus formation at 8 h to determine their size. (B) Quantification of WT and *pbgA* mutant focus formation at 8 h. (C) Low-magnification image of WT and *clsA* mutant foci and small plaque formation at 16 h to determine their size. (D) Quantification of WT and *clsA* mutant foci and small plaque formation at 16 h. Values that are significantly different (*P*  < 0.0001) by Student’s *t* test are indicated by a bar and four asterisks.

Intercellular spread of *S. flexneri* is a dynamic, multistep process (22); it requires the bacteria to polymerize host actin to move and penetrate neighboring cells (4), reactivate their T3SS to eject effector proteins into neighboring cell cytoplasm (51), and resolve the membrane protrusions into neighboring cells (9). To determine specifically at which point during intercellular spread the *clsA* and *pbgA* mutants have a defect, we used infection of HT-29 intestinal cells, which allowed us to employ time-lapse confocal microscopy to monitor cell-to-cell spread over an extended period of time (52). The bacteria are easily visualized in HT-29 cells, but they grow and spread more slowly in this cell line than in HeLa cells. Thus, longer time periods were used for the spread analysis. First, cell-to-cell spread was quantified by using computer-assisted image analysis to measure the areas of the infected foci in cellular monolayers (Fig. 5A). After 8 h, the *pbgA* mutant, but not the complemented strain, showed a significant decrease in the area of intercellular spread compared to the WT (Fig. 5B), indicating that outer membrane cardiolipin is required for *S. flexneri* intercellular spread early in infection. The *clsA* mutant also showed a defect in spread, although a significant decrease in the focus area in HT-29 cells was not detected until 8 to 16 h of infection (Fig. 5C and D). The difference in the rate of spread of the *clsA* mutant compared to that of the *pbgA* mutant may be due to increased PG in the outer membrane of the *clsA* mutant that partially compensates for the loss of cardiolipin, which does not occur in the strain lacking PbgA.

### Cardiolipin synthesis is required for proper intracellular division of *S. flexneri*.

**FIG 6:**
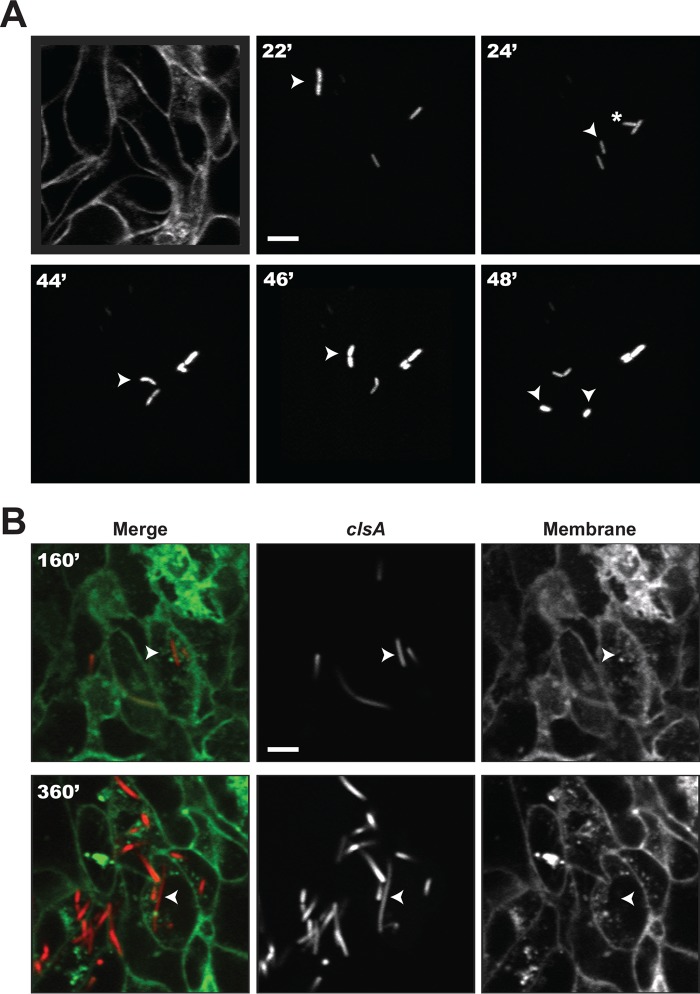
Visualization of *clsA* mutant filamentation in HT-29 cells. (A) Basal Z-section slice of the HT-29 monolayer footprint prior to infection and early infection tracking of a normally growing bacterium. Arrowhead tracking of a bacterium about to divide at 22 min (22′), which gives rise to two bacteria shown at 24 min, indicated by an arrowhead and asterisk. The arrowhead was tracked until the next cell division shown at 48 min. (B) Late infection tracking of one cytosolic filamenting bacterium at 160 and 360 min. Bars = 5 μm.

Using the HT-29 intestinal cell line model, we analyzed the timing of cell-to-cell spread of the *clsA* mutant by time-lapse confocal microscopy (9). HT-29 cell monolayers expressing plasma membrane-targeted yellow fluorescent protein (YFP) were infected with *S. flexneri* expressing isopropyl-β-d-thiogalactopyranoside (IPTG)-inducible cyan fluorescent protein (CFP), and individual bacteria were tracked. The wild-type strain showed rapid cell division, motility and spread to adjacent cells (Movie S1). The behaviour of the *clsA* mutant (Movie S2) was similar to that of the wild type at the early time points. It was motile in the initially infected cell and had normal intracellular growth (Fig. 6A). However, after the initial rounds of replication, the *clsA* mutant began to form filaments (Fig. 6), and there was a loss of motility (Movie S2). Some of the *clsA* mutant cells formed protrusions that were unable to form vacuoles and retracted back into the cell (Movie S2). Thus, cardiolipin is required in the membrane of *S. flexneri* for proper cell division after extended intracellular growth.

10.1128/mBio.01199-17.7 MOVIE S1  Wild-type dissemination. Time-lapse confocal microscopy of wild-type *S. flexneri* intercellular spread. Time points are separated by 2 min, for 6 h beginning 2 h postinfection. Download MOVIE S1, MPG file, 1.6 MB. Copyright © 2017 Rossi et al. 2017 Rossi et al. This content is distributed under the terms of the Creative Commons Attribution 4.0 International license.

10.1128/mBio.01199-17.8 MOVIE S2  *clsA* dissemination. Time-lapse confocal microscopy of *clsA* mutant intercellular spread. Time points are separated by 2 min, for 6 h beginning 2 h postinfection. Download MOVIE S2, MPG file, 0.9 MB. Copyright © 2017 Rossi et al. 2017 Rossi et al. This content is distributed under the terms of the Creative Commons Attribution 4.0 International license.

### The transport of cardiolipin to *S. flexneri*’s outer membrane is not required for intracellular growth.

To define more precisely whether the defect in intracellular replication in the cardiolipin synthesis mutant (*clsA*) was due to lack of cardiolipin in the inner or outer membrane, we also performed time-lapse microscopy on the *pbgA* mutant, which has cardiolipin in the inner membrane, but not in the outer membrane (Movie S3). Unlike the *clsA* mutant, the *pbgA* mutant grew normally within the cytoplasm of HT-29 cells and showed no filamentation, indicating that cardiolipin is needed in the inner membrane for maintaining wild-type replication in the intracellular environment. However, the *pbgA* mutant was nonmotile inside the eukaryotic cell and did not spread intercellularly. Thus, cardiolipin or increased PG in the outer membrane promotes intracellular motility. For comparison, the *vpsC* mutant, which has altered phospholipids and lipid A modifications in the outer membrane (28), was analyzed (Movie S4). The *vpsC* mutant had an intracellular infection phenotype that was distinct from either of the cardiolipin mutants. It replicated normally and was motile, but its motility was less than the wild-type motility, and there was no spread to adjacent cells.

10.1128/mBio.01199-17.9 MOVIE S3  *pbgA* dissemination. Time-lapse confocal microscopy of *pbgA* mutant intercellular spread. Time points are separated by 2 min, for 6 h beginning 2 h postinfection. Download MOVIE S3, MPG file, 0.9 MB. Copyright © 2017 Rossi et al. 2017 Rossi et al. This content is distributed under the terms of the Creative Commons Attribution 4.0 International license.

10.1128/mBio.01199-17.10 MOVIE S4  *vpsC* dissemination. Time-lapse confocal microscopy of *vspC* mutant intercellular spread. Time points are separated by 2 min, for 6 h beginning 2 h postinfection. Download MOVIE S4, MOV file, 1.1 MB. Copyright © 2017 Rossi et al. 2017 Rossi et al. This content is distributed under the terms of the Creative Commons Attribution 4.0 International license.

### Unipolar IcsA localization requires the cardiolipin transporter PbgA.

**FIG 7:**
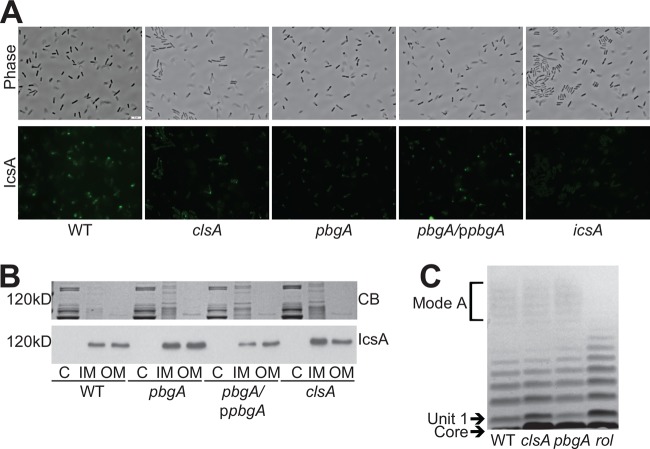
*pbgA* is required for unipolar IcsA localization. (A) Visualization of IcsA localization. Bacterial cultures were grown to mid-log phase (visualized by phase contrast microscopy), and IcsA was observed by indirect immunofluorescence (visualized by FITC). All images were captured with an exposure time of 1.5 s and processed in an identical manner. Bar = 5 μm. (B) Outer membrane IcsA levels. Bacteria were grown to mid-log phase. The membranes were fractionated using Sarkosyl membrane solubilization, resolved by (10%) SDS-PAGE, stained with Coomassie blue (CB) (same gel picture in Fig. 2B), and immunoblotted using either polyclonal anti-IcsA antisera. C, cytoplasm; IM, inner membrane; OM, outer membrane. (C) LPS structure of *clsA* and *pbgA* mutants. Bacteria were grown to mid-log phase. LPS was extracted, resolved by (4 to 12%) SDS-PAGE, and visualized by silver staining.

Because the *pbgA* mutant was nonmotile within the intracellular environment, it was likely that the defect was in the export or localization of IcsA. To determine whether PbgA was required for IcsA localization, we used indirect immunofluorescence directed against IcsA to visualize surface IcsA localization of *S. flexneri* grown *in vitro* (16). Compared to the WT, the *pbgA* mutant displayed very low levels of surface IcsA recognized by the antibody (Fig. 7A). Wild-type, unipolar localization of IcsA was restored with full-length *pbgA* on a plasmid (Fig. 7A). This suggested that the lack of cardiolipin within the outer membrane, the only known defect of the *pbgA* mutant, reduced localization of IcsA on the surface of the bacteria.

The *clsA* mutant, which also lacks cardiolipin in the outer membrane, retained motility during the initial stage of infection of HT-29 cells, and IcsA localization at the pole was detected in the *clsA* mutant, although it was reduced compared to the wild type (Fig. 7A). Therefore, if cardiolipin normally plays a role in IcsA localization in wild-type cells, the increase in outer membrane PG that occurs in the *clsA* mutant, but not the *pbgA* mutant, may be able to partially compensate for the loss of cardiolipin.

Although the anionic lipids cardiolipin and PG are known to be capable of localizing membrane proteins, the disruption in localization of IcsA could be indirect. Export of IcsA to the outer membrane (15) may be inefficient in the *pbgA* mutant, or mislocalization of IcsA could be due to disruption in LPS chain length (23) if the mutations affect LPS structure. To determine whether cardiolipin plays a role in efficient transport of IcsA to the outer membrane, we fractionated the inner and outer membranes of *S. flexneri* and examined the IcsA levels in each membrane using immunoblot analysis. We found that both the *clsA* and *pbgA* mutants had WT levels of IcsA within their outer membrane (Fig. 7B), indicating that cardiolipin synthesis or transport is not necessary for IcsA stability in the membrane or its export to the *S. flexneri* outer membrane. To determine whether the absence of cardiolipin disrupts the LPS of *S. flexneri*, we compared the LPS profiles of *clsA* and *pbgA* mutants to the wild-type LPS and to a previously characterized *rol* LPS mutant (23) by gel electrophoresis. The *clsA* and *pbgA* mutants did not have any detected differences in LPS chain lengths compared to the WT (Fig. 7C). Together, these data indicate that cardiolipin or compensatory levels of PG in the outer membrane are directly involved in the localization of IcsA on the surface.

## DISCUSSION

To cause disease, *S. flexneri* must efficiently invade colonic epithelial cells and spread intercellularly to neighboring cells. Penetration of the adjacent cellular membranes requires *S. flexneri* to move within the cytoplasm, and this movement is a direct result of IcsA-mediated actin polymerization (13). We have previously shown that outer membrane integrity, mediated by asymmetric distribution of phospholipids and lipid A structure, is required by *S. flexneri* during intercellular spread (28); however, the roles of specific phospholipids in *S. flexneri* pathogenesis have not been determined. In this study, we show that the phospholipid cardiolipin is required in the inner membrane of *S. flexneri* for proper cell division during intercellular spread (Fig. 6B), while outer membrane cardiolipin is associated with proper localization of IcsA and intercellular spread (Fig. 7A).

Cardiolipin is a large anionic phospholipid that makes up only 5 to 10% of *S. flexneri*’s membrane phospholipids (Fig. 1B). Much of what is currently known about cardiolipin’s role in biological membranes is the result of studies of the eukaryotic mitochondria, where cardiolipin makes up a large portion of the inner mitochondrial matrix to promote the localization and activity of electron transport proteins (36). Cardiolipin function is largely conserved in *E. coli*, where it is required for localization of high-energy electron transport (40) and osmotic proteins (42) within the bacterial inner membrane. Still, much remains unknown regarding cardiolipin’s role in bacterial membranes, and this is likely for two reasons. First, most enteric bacteria have three cardiolipin synthases, and all three must be inactivated in order to eliminate cardiolipin from the bacterial membrane (32); most studies thus far have been performed only on bacteria with reduced cardiolipin levels. Second, PG, which is similar to cardiolipin in that it also has a glycerol head group and is an anionic phospholipid, can interact with proteins in a similar manner and compensate for the lack of cardiolipin within the bacterial membrane (43). Thus, bacteria lacking cardiolipin do not display an *in vitro* growth phenotype.

To date, the roles of cardiolipin in bacterial pathogenesis have been identified in two pathogens, *S*. Typhimurium and *Moraxella catarrhalis*. In *S*. Typhimurium, cardiolipin is required in the outer membrane to provide membrane integrity during infection. Increased expression of *pbgA* and remodeling of the outer membrane occur in response to PhoPQ signaling during *Salmonella* infection (34). *Salmonella*, like *Shigella*, is an intracellular pathogen; however, *Salmonella* resides within lysosomes of macrophages, which may represent a more stressful environment than the cytoplasm. Since *S. flexneri* lacking cardiolipin does not have reduced membrane integrity when grown in the presence of DOC, and does not have an *in vivo* growth defect, it is unlikely that the role of cardiolipin in *S. flexneri* pathogenesis is maintenance of *S. flexneri* membrane integrity.

In *M. catarrhalis*, cardiolipin is required for proper bacterial attachment to human epithelial cells (53). It is hypothesized that cardiolipin is required for the localization or display of adhesion proteins. This may represent a similar function to cardiolipin’s role in *S. flexneri*, where cardiolipin in the outer membrane is important for the localization of the IcsA (Fig. 7A).

In *E. coli*, cardiolipin specifically localizes to the inner leaflet of the bacterial poles (39). This is because cardiolipin has a small glycerol head group and a large acyl region with four chains, giving the overall structure of cardiolipin a conical shape. Bacteria lacking cardiolipin do not have altered cell morphology, supporting the model that the conical shape of cardiolipin does not dictate the negative curvature of the poles. Rather, its localization at the poles is a consequence of its shape. Cardiolipin localizes to bacterial poles via diffusion, because of its natural tendency to destabilize planar membranes. In the absence of cardiolipin, PG localizes to the bacterial pole and can interact with polar proteins in the same manner but less efficiently than cardiolipin (43). We predict that in cardiolipin’s absence in *S. flexneri*, increased PG in the outer membrane can help localize IcsA to the pole; however, this localization does not appear to be as specific or as efficient as when cardiolipin is present. Polar IcsA localization is directed also by the outer leaflet LPS structure (23–25). Cardiolipin does not affect LPS structure, but it is not known whether the LPS structure affects outer membrane cardiolipin localization. It is possible that changes in the LPS structure disrupt polar localization of cardiolipin, indirectly causing the mislocalization of IcsA.

Cardiolipin may directly interact with IcsA, as has been shown for some proteins, to concentrate the protein at the pole. In the inner mitochondrial matrix, lysine residues on the surface of Drp1 interact with the glycerol head group of cardiolipin, helping it localize with cardiolipin (54). It is possible that positively charged residues on the surface of the IcsA beta barrel or the polar targeting (PT) domain identified in the N-terminal region of IcsA (55, 56) help IcsA localize with cardiolipin. Alternatively, cardiolipin could indirectly localize IcsA by interacting with the positively charged residues of other proteins known to aid in its localization and activity (57). For example, IcsA chaperone (15, 16, 58, 59) or secretion proteins (60) may interact with cardiolipin to direct insertion of IcsA into the outer membrane at the poles. These interactions would promote polar localization of IcsA to allow directed movement when actin polymerizes at the bacterial surface.

The loss of cardiolipin from the outer membrane without compensation by increased PG affects plaque formation by preventing IcsA localization. The effects of loss of cardiolipin from the inner membrane are less clear. The *clsA* mutant replicates normally *in vitro* and has no obvious defect early in infection of epithelial cells. However, longer exposure to the intracellular environment results in aberrant cell division and loss of motility. Studies of *E. coli* have shown that the cell division protein MinD and osmotic stress proteins associate with cardiolipin in the inner membrane (42, 43). Similar effects of cardiolipin on localization of inner membrane proteins in *S. flexneri* could cause the growth defects seen in the host cell cytoplasm.

## MATERIALS AND METHODS

### Bacterial strains and growth conditions.

Bacterial strains and plasmids used in this study are found in Table S2 in the supplemental material. All strains were maintained at −80°C in tryptic soy broth (TSB) containing 20% (vol/vol) glycerol. *E. coli* strains were grown on Luria-Bertani (LB) agar (1% tryptone, 0.5% yeast extract, 1% NaCl, 1.5% agar [wt/vol]) at 37°C, and single colonies were selected and grown in LB broth at 37°C. *S. flexneri* strains were grown on TSB agar (TSB, 1.5% agar [wt/vol]) containing Congo red dye (0.01% [wt/vol]; Sigma) at 37°C. Congo red binding colonies (61), indicating a functional T3SS, were selected and grown in LB broth at 30°C for maintenance and were then subcultured 1:100 and grown at 37°C for assays. The following antibiotics were used at the indicated concentrations: kanamycin, 50 μg/ml; ampicillin, 25 μg/ml.

10.1128/mBio.01199-17.5 TABLE S2  Strains and plasmids used in this study. Superscript *a* indicates the genome or locus accession number. Download TABLE S2, DOCX file, 0.1 MB. Copyright © 2017 Rossi et al. 2017 Rossi et al. This content is distributed under the terms of the Creative Commons Attribution 4.0 International license.

### Construction of *S. flexneri* mutants.

The *S. flexneri clsA*, *clsB*, *clsC*, and *vpsC* mutants were created by bacteriophage P1 transduction of the Δ*clsA*::*kan*, Δ*clsB*::*kan*, Δ*clsC*::*kan*, and Δ*mlaD*::*kan* alleles from *E. coli* strains JW1241 (Keio Collection), JW0772 (Keio Collection), JW5150 (Keio Collection), and JW3160 (Keio Collection), respectively, into *S. flexneri* strain 2457T (62). Double and triple mutants were created by using a multistep procedure whereby the kanamycin resistance cassette used to create an existing mutation was removed using the plasmid pCP20 (63), followed by P1 transduction to mutate additional genes. An *S. flexneri pbgA* mutant was created using λ-Red-mediated recombination (64). A PCR product was generated by amplifying the kanamycin resistance cassette from pKD4 using primers pbgA-KO-F and pbgA-KO-R, which modified the wild-type *pbgA* gene by introducing a UGA termination codon in place of the codon for Y190 and replaces the downstream codons with a kanamycin resistance cassette (33, 64). This PCR product was introduced by electroporation into *E. coli* strain BW25113 expressing λ-Red recombinase from plasmid pKD46 (64), and recombinants were selected by kanamycin resistance. This mutation was then introduced into *S. flexneri* through bacteriophage P1 transduction. All mutations were verified via PCR.

### Construction of plasmids.

Plasmids expressing *clsA* and *pbgA* (p*clsA* and p*pbgA*, respectively) were constructed by amplifying the wild-type loci containing the native promoter region from *S. flexneri* strain 2457T and ligating the PCR product into the SmaI site of pWKS30 (65). Primers used in this study are listed in Table S3. Primers clsA-F and clsA-R were used to amplify *clsA*, and primers pbgA-F and yeM-R were used to amplify *pbgA*, using *S. flexneri* genome as the template (66). Constructed plasmids were sequenced at the University of Texas at Austin DNA sequencing facility using an ABI 3130 sequencer (Applied Biosystems).

10.1128/mBio.01199-17.6 TABLE S3  Primers used in this study. Download TABLE S3, DOCX file, 0.1 MB. Copyright © 2017 Rossi et al. 2017 Rossi et al. This content is distributed under the terms of the Creative Commons Attribution 4.0 International license.

### Cell culture media and growth conditions.

Henle cells (intestine 407; ATCC CCL-6) were cultured in minimal essential medium (MEM) (Gibco) containing 10% (vol/vol) fetal bovine serum (FBS) (Gibco), 10% (wt/vol) Bacto tryptone phosphate broth (Difco), 1× nonessential amino acids (Gibco), and 2 mM glutamine. Colorectal cells (HT-29; ATCC HTB-38) were cultured in McCoy’s 5A medium (Gibco) supplemented with 10% (vol/vol) heat-inactivated FBS (Invitrogen). Henle and HT-29 cells were incubated at 37°C with 95% air and 5% CO_2_. Gentamicin was used at a final concentration of 40 μg/ml.

### Isolation and analysis of phospholipid species.

Bacteria were grown in LB to an optical density at 650 nm (OD_650_) of ~0.5 (mid-log phase) or as indicated. Phospholipids were extracted by the method of Bligh and Dyer (44). Phospholipids were then spotted onto a silica gel 60 (Millipore) thin-layer chromatography (TLC) plate and separated by TLC using a chloroform-methanol-acetic acid (65:25:10, vol/vol/vol) solvent system (67). Phospholipids were detected by spraying TLC plates with molybdenum blue spray reagent (Sigma). The area of species was quantified using ImageJ (68) to determine the percentage of each phospholipid in the sample.

Inner and outer membranes were isolated by pelleting mid-log-phase bacteria at 13,000 × *g* for 10 min, resuspending in buffer containing 10 mM Na_2_HPO_4_ and 5 mM MgSO_4_, sonicating to induce cell lysis, and centrifuging at 13,000 × *g* for 20 min to remove cell debris. The supernatant was then centrifuged at 135,000 × *g* for 40 min to isolate the total membranes. Total membranes were resuspended in 1.0% (wt/vol) Sarkosyl using a blunt needle and incubated at room temperature for 20 min. Following centrifugation at 135,000 × *g* for 40 min, the inner membranes remained in the supernatant, while the outer membranes were pelleted. The pelleted outer membranes were resuspended in fresh 1.0% (wt/vol) Sarkosyl and used for downstream assays.

### Cell culture assays.

Plaque assays were performed as previously described (47). Briefly, bacteria were grown to an OD_650_ of ~0.5. Approximately 10^4^ CFU of bacteria were added to a confluent monolayer of Henle cells in 35-mm, 6-well polystyrene plates (Corning) and centrifuged for 10 min at 1,000 × *g*. The plates were incubated for 60 min, and the monolayers were washed four times with phosphate-buffered saline (PBS-D) (1.98 g KCl, 8 g NaCl, 0.02 g KH_2_PO_4_, 1.39 g K_2_HPO_4_). The medium was then replaced with MEM containing gentamicin and 0.45% (wt/vol) glucose, and the plates were then incubated for 24 h, after which the medium was replaced with MEM containing only gentamicin, and the plates were incubated for an additional 48 h. The monolayers were washed with PBS-D and stained with Wright-Giemsa stain for visualization.

Cell-to-cell spread assays were performed as previously described (28). Briefly, bacteria were grown to an OD_650_ of ~0.5. Approximately 10^7^ CFU of bacteria were added to a confluent monolayer of Henle cells in 35-mm, 6-well polystyrene plates (Corning) and centrifuged for 10 min at 1,000 × *g*. The plates were incubated for 30 min, and the monolayers were then washed four times with PBS-D. The medium was replaced with MEM containing gentamicin, and the plates were incubated for an additional 4 h. The monolayers were washed with PBS-D and stained with Wright-Giemsa stain. Henle cells were scored positive for spread if they contained three or more *S. flexneri* cells and if adjacent Henle cells also contained three or more *S. flexneri* cells. One hundred Henle cells per well were counted.

### Fluorescence microscopy techniques.

The size of infection foci formed in plasma membrane-YFP-expressing HT-29 cells grown in McCoy’s medium (Gibco, Life Technologies) and infected with the listed CFP-expressing *S. flexneri* strains was determined in a 96-well plate format (catalog no. 3904; Corning). After fixation, the plates were imaged using the ImageXpress Micro imaging system (Molecular Devices), and image analysis for focus size determination was performed with the ImageXpress imaging software (Molecular Devices) as previously described (52).

Bacterial dissemination was monitored using time-lapse confocal microscopy. Plasma membrane-YFP-expressing HT-29 cells were grown in McCoy’s medium in eight-well chambers (Lab-Tek II [catalog no. 155409; Thermo Fisher Scientific]) at 37°C in 5% CO_2_. Cells were infected with the listed CFP-expressing *S. flexneri* strains and imaged with a Leica DMI 8 spinning-disc confocal microscope driven by the iQ software (Andor). Z-stacks were captured 2 h postinfection every 2 min for 6 h. The corresponding movies were generated with the Imaris software (Bitplane).

IcsA localization was monitored as previously described (16). Briefly, bacteria were grown to mid-logarithmic phase and fixed in 4% (vol/vol) paraformaldehyde in PBS. The cells were then labeled by indirect immunofluorescence, using rabbit polyclonal antibody against IcsA (rabbit 35) diluted 1:100, provided by Edwin Oaks (Walter Reed Army Institute of Research), and a fluorescent isothiocyanate-conjugated goat anti-rabbit secondary antibody diluted 1:100 (15). Images were acquired using a DP73 digital microscope camera (Olympus) and processed using cellSens software (Olympus). Images were postprocessed with Lightroom (Adobe) to increase contrast. All images were processed using the same settings.

### SDS-PAGE and immunoblotting.

Inner and outer membranes were isolated by membrane fractionation as described above and resuspended in Laemmli SDS sample buffer (5% β-mercaptoethanol, 3% [wt/vol] SDS, 10% glycerol, 0.02% bromophenol blue, 63 mM Tris-Cl [pH 6.8]) (69), and boiled for 5 min. Samples were electrophoresed in quadruplicate (10%) SDS-polyacrylamide gels for separation. Proteins from three gels were transferred to a 0.45-μm-pore-size nitrocellulose membrane (GE Healthcare) and incubated with either rabbit polyclonal anti-IcsA antibody (Edwin Oaks, Walter Reed Army Institute of Research) diluted 1:10,000, rabbit polyclonal anti-SecA antibody (Donald Oliver, Wesleyan University) diluted 1:10,000, or rabbit polyclonal anti-OmpA antibody (Donald Oliver, Wesleyan University) diluted 1:5,000. Proteins were detected using horseradish peroxidase-conjugated goat anti-rabbit antibody (diluted 1:5,000). Signal was detected by developing the blot with Pierce ECL detection kit (Thermo Fisher). Proteins from the fourth gel were visualized by Coomassie brilliant blue staining and used to assess equal loading of samples for immunoblotting.

### Analysis of lipopolysaccharides.

LPS was isolated and analyzed as previously described (70). Briefly, bacteria were grown at 37°C to an OD_650_ of ~0.5, and the equivalent of OD_650_ of 1 was pelleted and resuspended in Laemmli SDS-PAGE sample buffer (69). Samples were then boiled for 10 min, cooled to room temperature, and treated with 25 μg proteinase K for 1 h at 55°C. LPS was visualized by (4 to 12%) SDS-PAGE (Bolt Bis-Tris Plus; Invitrogen) and subsequent staining of the LPS with silver stain as follows. The gels were fixed in 40% isopropanol and 5% acetic acid, oxidized with 0.7% periodic acid, stained with 20% silver nitrate, and developed using 50 μg/ml citric acid.

10.1128/mBio.01199-17.1 TEXT S1  Supplemental Materials and Methods used for supplemental experiments. Download TEXT S1, DOCX file, 0.02 MB. Copyright © 2017 Rossi et al. 2017 Rossi et al. This content is distributed under the terms of the Creative Commons Attribution 4.0 International license.
