# Supplementary material for: Cardiolipin Synthesis and Outer Membrane Localization Are Required for Shigella flexneri Virulence
Source: mBio. 2017 Aug 29;8(4):e01199-17. doi: 10.1128/mBio.01199-17 (PMC5574711; doi:10.1128/mBio.01199-17)
Supplement: FIG S1 [file mbo004173433sf1.docx]

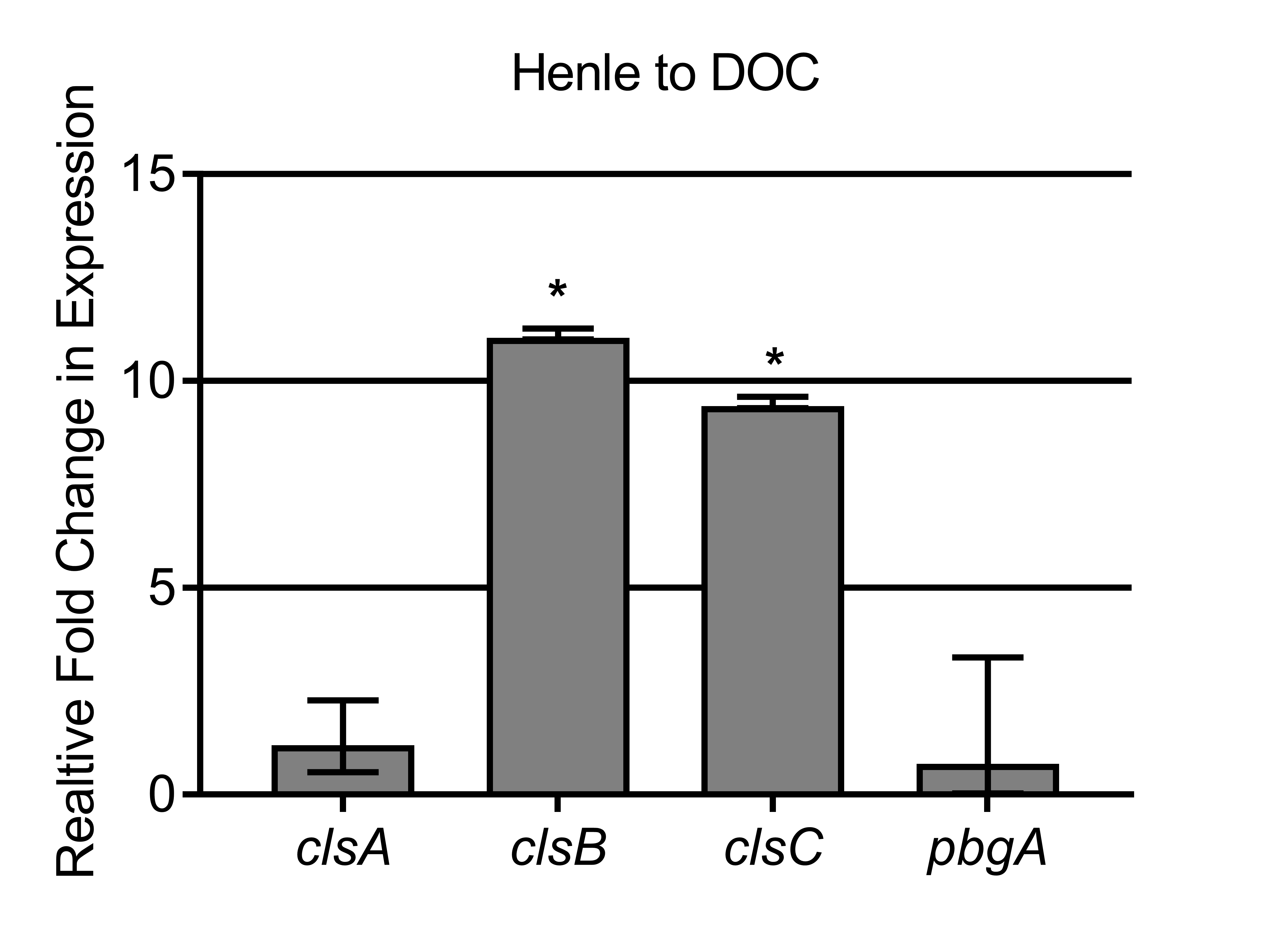
**Fig. S1.** mRNA levels of *clsB* and *clsC* increase during intracellular growth, as determined by quantitative real-time PCR. Bacteria were subcultured 1:100 into LB + 0.01% DOC and grown to midlog phase. Cultures were divided, and RNA from approximatly10^8^ CFU was isolated and used to determine extracellular message levels, and semiconfluent Henle-407 monolayers were infected with approximately 10^8^ CFU. RNA was isolated 4 hours post infection and used to determine intracellular mRNA levels. Threshold cycle (*C_T_*) values were normalized against those for *accD,* analysis was performed using the *ΔΔC_T_* approach and are shown relative to the extracellular level set to 1. (*) *P*-value of <0.05 compared to the extracellular expression levels (by Student’s t-test).
