# Supplementary material for: Cardiolipin Synthesis and Outer Membrane Localization Are Required for Shigella flexneri Virulence
Source: mBio. 2017 Aug 29;8(4):e01199-17. doi: 10.1128/mBio.01199-17 (PMC5574711; doi:10.1128/mBio.01199-17)
Supplement: FIG S2 [file mbo004173433sf2.docx]

**Fig. S2.** *clsA* and *pbgA* mutant strains do not exhibit growth sensitivity to DOC. (A) Bacteria were subcultured 1:100 into LB (A), or LB containing 0.1% DOC (B), and grown into stationary phase. Data shown is representative of three biological replicates.
