# Supplementary material for: Cardiolipin Synthesis and Outer Membrane Localization Are Required for Shigella flexneri Virulence
Source: mBio. 2017 Aug 29;8(4):e01199-17. doi: 10.1128/mBio.01199-17 (PMC5574711; doi:10.1128/mBio.01199-17)
Supplement: TABLE S1 [file mbo004173433st1.docx]

**Table S1.** Invasion and intracellular growth rates of *clsA* and *pbgA* mutants. ***^a^*** Percent of Henle cells that contained 3 or more intracellular bacteria. The data represent mean values of three biological replicates with standard deviation. *^b^* Intracellular doubling time of bacteria between 1 and 3 hours of intracellular growth. The numbers of intracellular bacteria were determined by lysing Henle monolayers, followed by plating of the lysate dilutions. Data represent the mean values of three biological replicates and standard deviations. Compared to wild type, none of the mutants had a statistical difference in invasion or intracellular doubling time by Student’s t-test, *P*-value <0.05.

| **Strain** | **Invasion (%)*^a^*** | | **Intracellular doubling time (min)*^b^*** |
| --- | --- | --- | --- |
|  | **- DOC** | **+ DOC** |  |
| WT | 30 ± 3 | 66 ± 3 | 31 ± 7 |
| *clsA* | 29 ± 8 | 68 ± 3 | 27 ± 7 |
| *pbgA* | 29 ± 2 | 57 ± 5 | 23 ± 3 |
| *icsA* | 29 ± 5 | 58 ± 11 | 30 ± 4 |
