# Supplementary material for: Cardiolipin Synthesis and Outer Membrane Localization Are Required for Shigella flexneri Virulence
Source: mBio. 2017 Aug 29;8(4):e01199-17. doi: 10.1128/mBio.01199-17 (PMC5574711; doi:10.1128/mBio.01199-17)
Supplement: TABLE S2 [file mbo004173433st2.docx]

**Table S2.** Strains and plasmids used in this study. *^a^* Genome or loci ascension number.

| **Strain or plasmid** | **Description** | **Reference** |
| --- | --- | --- |
| ***E. coli*** |  |  |
| Top10 | Cloning strain | Thermo Fisher Scientific |
| W3110 | Wild Type | (1) |
| BW25113 | Wild Type | (2) |
| JW1241 | BW25113 *Δcls*::*kan* | Keio Collection (3) |
| JW0772 | BW25113 *ΔybhO*::*kan* | Keio Collection (3) |
| JW5150 | BW25113 *ΔymdC*::*kan* | Keio Collection (3) |
| JW3160 | BW25113 *ΔmlaD*::*kan* | Keio Collection (3) |
| *E.C.-pbgA* | BW25113 *ΔyejM190-586*::*kan* | This study (4) |
| ***S. flexneri* 2a** |  |  |
| WT | 2457T Wild Type, serotype 2a (AE014073.1)*^a^* | Walter Reed Army Institute of Research (5) |
| *clsA* | 2457T *Δcls*::*kan* (AAP16748.1) | This study |
| *clsB* | 2457T *ΔybhO*::*kan* (AAP16251.1) | This study |
| *clsC* | 2457T *ΔymdC*::*kan* (AAP16548.1) | This study |
| *clsAB* | 2457T *Δcls*::FRT*, ΔybhO*::*kan* | This study |
| *clsAC* | 2457T *Δcls*::FRT*, ΔymdC*::*kan* | This study |
| *clsBC* | 2457T *ΔymdC*::FRT*, ΔybhO*::*kan* | This study |
| *clsABC* | 2457T *Δcls*::FRT*, ΔymdC*::FRT*, ΔybhO*::*kan* | This study |
| *pbgA* | 2457T *ΔyejM190-586*::*kan* (AAP17611.1) | This study |
| *icsA* | 2457T *icsA*::E2-Tn5 (AAL72293.1) | H. Marman |
| *vpsC* | 2457T, *ΔvpsC*::*kan* (AAP18513.1) | This study (6) |
| *rol* | SA100, serotype 2a *Δrol*::*kan* | (7) |
| **Plasmids** |  |  |
| pKD4 | *kan* cassette template | (2) |
| pKD46 | Ts Red recombinase | (2) |
| pCP20 | Ts Flp recombinase | (8) |
| pWKS30 | Low-copy Expression vector | (9) |
| p*clsA* | *clsA* in pWKS30 | This study |
| p*pbgA* | *pbgA* in pWKS30 | This study |
| p*cfp* | *cfp*-expressing plasmid | (10) |
| p*yfp* | *yfp*-expressing plasmid | (10) |

1. **Oliver PM**, **Crooks JA**, **Leidl M**, **Yoon EJ**, **Saghatelian A**, **Weibel DB**. 2014. Localization of anionic phospholipids in *Escherichia coli* cells. J Bacteriol **196**:3386–3398.

2. **Datsenko KA**, **Wanner BL**. 2000. One-step inactivation of chromosomal genes in *Escherichia coli* K-12 using PCR products. Proc Natl Acad Sci USA **97**:6640–6645.

3. **Baba T**, **Ara T**, **Hasegawa M**, **Takai Y**, **Okumura Y**, **Baba M**, **Datsenko KA**, **Tomita M**, **Wanner BL**, **Mori H**. 2006. Construction of *Escherichia coli* K-12 in-frame, single-gene knockout mutants: the Keio collection. Mol Syst Biol **2**:2006.0008.

4. **De Lay NR**, **Cronan JE**. 2008. Genetic interaction between the *Escherichia coli* AcpT phosphopantetheinyl transferase and the YejM inner membrane protein. Genetics **178**:1327–1337.

5. **Wei J**, **Goldberg MB**, **Burland V**, **Venkatesan MM**, **Deng W**, **Fournier G**, **Mayhew GF**, **Plunkett G**, **Rose DJ**, **Darling A**, **Mau B**, **Perna NT**, **Payne SM**, **Runyen-Janecky LJ**, **Zhou S**, **Schwartz DC**, **Blattner FR**. 2003. Complete genome sequence and comparative genomics of *Shigella flexneri* serotype 2a strain 2457T. Infect and Immun **71**:2775–2786.

6. **Carpenter CD**, **Cooley BJ**, **Needham BD**, **Fisher CR**, **Trent MS**, **Gordon V**, **Payne SM**. 2014. The Vps/VacJ ABC transporter is required for intercellular spread of *Shigella flexneri*. Infect and Immun **82**:660–669.

7. **Hong M**, **Payne SM**. 1997. Effect of mutations in *Shigella flexneri* chromosomal and plasmid-encoded lipopolysaccharide genes on invasion and serum resistance. Mol Microbio **24**:779–791.

8. **Cherepanov PP**, **Wackernagel W**. 1995. Gene disruption in *Escherichia coli*: TcR and KmR cassettes with the option of Flp-catalyzed excision of the antibiotic-resistance determinant. Gene **158**:9–14.

9. **Wang RF**, **Kushner SR**. 1991. Construction of versatile low-copy-number vectors for cloning, sequencing and gene expression in *Escherichia coli*. Gene **100**:195–199.

10. **Dragoi A-M**, **Agaisse H**. 2014. The serine/threonine kinase STK11 promotes *Shigella flexneri* dissemination through establishment of cell-cell contacts competent for tyrosine kinase signaling. Infect and Immun **82**:4447–4457.
