# Supplementary material for: Cardiolipin Synthesis and Outer Membrane Localization Are Required for Shigella flexneri Virulence
Source: mBio. 2017 Aug 29;8(4):e01199-17. doi: 10.1128/mBio.01199-17 (PMC5574711; doi:10.1128/mBio.01199-17)
Supplement: TABLE S3 [file mbo004173433st3.docx]

**Table S3.** Primers used in this study

| **Primer** | **Sequence (5’ to 3’)** |
| --- | --- |
| clsA-F | GCTGACAGTAAAGAATCGGC |
| clsA-R | ATCGTAGGCCTGCTCAAGCG |
| pbgA-KO-F | CCGCATTCTTATTTATCGCCTTTATCGCCTCGCATGTGGTGTATATCTGAGTGTAGGCTGGAGCTGCTTC |
| pbgA-KO-R | TTCCACACCGATTGCAAGTAAGATATTTCGCTAACTGATTTATAATTAATCATATGAATATCCTCCTTAG |
| pbgA-F | TAACGAGAATGATTTAACGC |
| pbgA-R | ATACTACCGTTTTCCACACC |
| RT-accD-F | ATGGCGAAACTTGCAGAAAC |
| RT-accD-R | AAGCACTGATGTCGCTGATG |
| RT-clsA-F | AGCAACGGGCTGAAGAAGTA |
| RT-clsA-R | AGGTTTTGGTGCAGACCTTG |
| RT-clsB-F | CGTACCGCGTAATCCTGTTT |
| RT-clsB-R | TTATTGGCGGGCTGAATTAC |
| RT-clsC-F | GCCGGATGCACAATAAAAGT |
| RT-clsC-R | GGCCTATTGCCATGACATCT |
| RT-pbgA-F | ATTCTGCTTTCGCTCGTCAT |
| RT-pbgA-R | CTGAAATGGCCGATAATGCT |
