# Supplementary material for: Cardiolipin Synthesis and Outer Membrane Localization Are Required for Shigella flexneri Virulence
Source: mBio. 2017 Aug 29;8(4):e01199-17. doi: 10.1128/mBio.01199-17 (PMC5574711; doi:10.1128/mBio.01199-17)
Supplement: TEXT S1 [file mbo004173433s1.docx]

**SUPPLEMENTAL MATERIALS AND METHODS**

**Quantative PCR (qPCR):** was performed as previously described . Briefly, CR^+^ colonies were grown overnight at 30°C in LB with aerating and then diluted 1:100 into LB+ 0.01% DOC and grown at 37°C with aeration to an OD_650_ of ~ 0.7. 1 X 10^8^. Using RNA-BEE (Tel-Test, Inc.), RNA was harvested from the cells and was followed by DNas I (Life Technologies) treatment to removed genomic DNA. Intracellular RNA was isolated by infecting semiconfluent Henle-407 monolayers with approximately 10^8^ CFU grown to midlog in LB + 0.01% DOC and 4 hours post infection monolayers were treated with RNA-BEE and treated in identical manner as described above. cDNA was then generated using Superscript III reverse transcriptase (Life Technologies), and Power SYBR green (Life Technologies) chemistry was used amplify and detect targets. Threshold cycle (*C_T_*) values were normalized against those for *accD* and analysis was performed using the *ΔΔC_T_* approach. Primers used for qPCR are listed in Table S2, indicated by RT in their primer name.

**Invasion assays:** were performed as previously described (1). Briefly, bacteria were grown to an OD_650_ of ~ 0.5. Approximately 10^8^ CFU of bacteria were added to a semi-confluent monolayer of Henle cells in 35 mm, 6-well, polystyrene plates (Corning) and centrifuged for 10 min at 1,000 X *g*. Plates were incubated for 30 min and monolayers were washed 4 times PBS-D. The media was replaced with MEM containing gentamicin, and the plates were incubated for an additional 40 min. Monolayers were washed with PBS-D and stained with Wright-Giemsa stain (Camco). Monolayers were visualized using bright-field microscopy at 1,000 X magnification. Henle cells were scored positive for invasion if they contained 3 or more *S. flexneri* cells, 300 Henle cells per well were counted.

**Intracellular growth rates:** were measured by growing bacteria in LB containing 0.1% DOC to an OD_650_ of ~ 0.5. Approximately 5 X 10^8^ CFU of bacteria were added to a confluent monolayer of Henle cells in 22.1 mm, 12-well, polystyrene plates (Corning) and centrifuged for 10 min at 1,000 X *g*. Plates were then incubated for 30 min. Monolayers were then washed 4 times with PBS-D, and media was replaced with MEM containing gentamicin. Monolayers were washed with PBS-D after 60 min and 180 min, and lysed using 1.0% DOC. Lysate were diluted and plated on TSB agar, grown overnight at 37°C, and colonies were counted. The number of bacteria recovered at 60 min and 180 min post infection were used to calculated the doubling time.

**1. Hale TL, Formal SB.** 1981. Protein synthesis in HeLa or Henle 407 cells infected with *Shigella dysenteriae* 1, *Shigella flexneri* 2a, or *Salmonella typhimurium* W118. Infect and Immun **32**:137–144.
